# Comprehensive Analysis and Biological Characterization of Venom Components from Solitary Scoliid Wasp *Campsomeriella annulata annulata*

**DOI:** 10.3390/toxins13120885

**Published:** 2021-12-10

**Authors:** Carlos Alberto-Silva, Fernanda Calheta Vieira Portaro, Roberto Tadashi Kodama, Halyne Queiroz Pantaleão, Hidetoshi Inagaki, Ken-ichi Nihei, Katsuhiro Konno

**Affiliations:** 1 Experimental Morphophysiology Laboratory, Natural and Humanities Sciences Center, Federal University of ABC (UFABC), São Bernardo do Campo 09606-070, SP, Brazil; halynequeiroz.p@gmail.com; 2 Structure and Functions of Biomolecules Laboratory, Butantan Institute, São Paulo 05503-900, SP, Brazil; fernanda.portaro@butantan.gov.br (F.C.V.P.); pararoberval@gmail.com (R.T.K.); 3 Biomedical Research Institute, National Institute of Advanced Industrial Science and Technology (AIST), 1-1-1 Higashi, Tsukuba 305-8566, Ibaraki, Japan; h-inagaki@aist.go.jp; 4 School of Agriculture, Utsunomiya University, Utsunomiya 321-8505, Tochigi, Japan; nihei98@cc.utsunomiya-u.ac.jp; 5 Institute of Natural Medicine, University of Toyama, Toyama 930-0194, Toyama, Japan

**Keywords:** solitary scoliid wasp, venom, comprehensive analysis, LC-MS, bradykinin-related peptide, linear α-helical peptide

## Abstract

Venoms of solitary wasps are utilized for prey capture (insects and spiders), paralyzing them with a stinger injection to be offered as food for their larvae. Thus, the identification and characterization of the components of solitary wasp venoms can have biotechnological application. In the present study, the venom components profile of a solitary scoliid wasp, *Campsomeriella annulata annulata*, was investigated through a comprehensive analysis using LC-MS and -MS/MS. Online mass fingerprinting revealed that the venom extract contains 138 components, and MS/MS analysis identified 44 complete sequences of the peptide components. The peptides are broadly divided into two classes: bradykinin-related peptides, and linear α-helical peptides. Among the components of the first class, the two main peptides, α-campsomerin (PRLRRLTGLSPLR) and β-campsomerin (PRLRRLTGLSPLRAP), had their biological activities evaluated. Both peptides had no effects on metallopeptidases [human neprilysin (NEP) and angiotensin-converting enzyme (ACE)] and acetylcholinesterase (AChE), and had no cytotoxic effects. Studies with PC12 neuronal cells showed that only α-campsomerin was able to enhance cell viability, while β-campsomerin had no effect. It is noteworthy that the only difference between the primary structures from these peptides is the presence of the AP extension at the C-terminus of β-campsomerin, compared to α-campsomerin. Among the linear α-helical peptides, annulatin (ISEALKSIIVG-NH_2_) was evaluated for its biological activities. Annulatin showed histamine releasing activity from mast cells and low hemolytic activity, but no antimicrobial activities against all microbes tested were observed. Thus, in addition to providing unprecedented information on the whole components, the three peptides selected for the study suggest that molecules present in solitary scoliid wasp venoms may have interesting biological activities.

## 1. Introduction

Arthropod venoms have been a subject of toxinological and pharmacological investigation, which have revealed that venoms are a rich source of pharmacologically and medically useful peptides [1]. Among them, arachnid (scorpions and spiders) venoms are the best studied, and a variety of peptides that could be developed for pharmacological tools, and medical and agricultural applications have been found [2,3,4,5]. Hymenopteran insects (bees and wasps) are also arthropods, and their venoms are another rich source of bioactive peptides (melittin, mastoparan, and waspkinins) and proteins (phospholipase C) [6,7].

Solitary wasps belong to the Hymenoptera order, but, in contrast to social wasps and bees, their venoms, and components, have been much less investigated. That may be due to their solitary lifestyle, living alone [8], and accordingly, making it difficult to collect enough venom (large numbers of wasp individuals) for chemical and pharmacological analyses. In recent years, however, toxinological and pharmacological investigation of solitary wasp venoms has advanced considerably, mainly due to remarkable progress of analytical methods using mass spectrometry [9,10,11,12,13,14].

We have surveyed solitary wasp venoms from Japan for the last decades, with interest in neuroactive substances contained in their venoms. Venoms of solitary wasps are utilized for prey capture (insects and spiders), paralyzing them with a stinger injection to be offered as food for their larvae. [8]. Therefore, solitary wasp venoms may contain neurotoxins and/or neuroactive molecules as important components. In fact, we found novel neurotoxins, pompilidotoxins (PMTXs) in spider wasp venoms [15], blocking both mammalian and insect sodium-channels [16,17]. Another neurotoxin, named Sa12b, is an FMRFamide-like peptide in sphecid wasp venoms, and inhibits acid-sensing ion channels (ASICs) [18]. In addition to these neurotoxins, we have found other types of bioactive peptides: antimicrobial peptides [19,20]; bradykinin-related peptides [21]; FMRFamide-like peptides [22]. Thus, our studies of solitary wasp venom components revealed not only the presence of neuroactive peptides, but also a variety of bioactive peptides [23].

Scoliid wasp is one of the groups of solitary wasps, belonging to the Scoliidae family, which hunt and sting beetle larva under the ground. Scoliid wasp venom components were investigated for the first time in 1987. From the scoliid wasp venoms of *Colpa interrupta* and *Megascolia flavifrons*, from Europe, two kinins, threonine^6^-bradykinin (Thr^6^-BK) and megascoliakinin, were isolated and pharmacologically characterized [24,25]. These kinins irreversibly block the synaptic transmission of the nicotinic acetylcholine receptor (nAChR) in the insect central nervous system [25,26]. Thr^6^-BK was identified in three scoliid wasp venoms from Japan through screening with MALDI-TOF MS [27]. Recently, we investigated the venom components of another Japanese scoliid wasp, *Scolia decorate ventralis*, and found a novel neuroprotective peptide, β-scoliidine, in a cellular stress model based on the H_2_O_2_-induced oxidative stress, which stimulates the excessive production of reactive oxygen species (ROS) [28].

The solitary scoliid wasp *Campsomeriella annulata annulata* is one of three species with Thr^6^-BK identified in their venom [27]. In this study, we investigated the venom components of this wasp in detail. The component profile was attained by comprehensive LC-MS and MS/MS analysis of crude venom extracts, which identified small molecules (amino acids, biogenic amines and nucleic acids), and many short peptide sequences. Two major structural types of peptides, bradykinin-related peptides, and linear α-helical peptides were identified, from which three novel peptides (α-campsomerin, β-campsomerin, and annulatin) were synthesized and biologically evaluated. Regarding bradykinin-related peptides, only α-campsomerin showed cell viability potentiating effect in neuronal PC12 cells, whereas β-campsomerin had no effect. Moreover, despite their structural resemblance to another neuroprotective peptide from the *Scolia decorata ventralis* wasp, different concentrations of two peptides did not show neuroprotective benefits against H_2_O_2_-induced oxidative stress in PC12 neuronal cells. Annulatin, classified as linear α-helical peptides, showed histamine releasing activity from mast cells. This is the first case that linear α-helical peptides were found in scoliid wasp venoms.

## 2. Results

### 2.1. Comprehensive Analysis of Venom Extract from Campsomeriella annulata annulata

#### 2.1.1. On-Line Mass Fingerprinting by LC-MS

**Figure 1:**
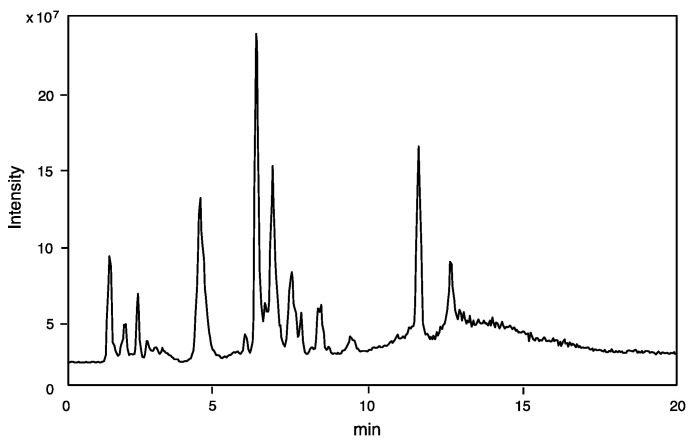
TIC profile from LC-ESI-MS of venom extracts of *Campsomeriella annulata annulata* by reverse-phase HPLC using CAPCELL PAK C_18_ (1.5 × 150 mm) with linear gradient of 5–65% CH_3_CN/H_2_O/0.1% formic acid over 20 min at flow rate of 200 μL/min.

The component profile (number of components and its molecular mass determination) was obtained by LC-ESI-MS analysis of the crude venom extract. Only 10% of the amount of venom sac extracts from a single specimen was sufficient for mass fingerprinting and peptide sequencing by LC-ESI-MS analysis. The TIC is shown in Figure 1.

Online mass fingerprint was prepared from TIC by “virtual fractionation”, collecting MS spectra from certain range of retention time (fractions), and then the molecular mass was analyzed in each fraction. The results are summarized in Table 1. A total of 138 components were found from 20 virtual fractions with a molecular mass range of *m*/*z* 90 to 6300. Most of the low molecular mass components of *m*/*z* 90–400 in the earlier fractions (Fr. 1–2, RT 0.8–2.0) may be small molecules (free amino acids, biogenic amines, and nucleic acids). Those in higher mass range and the later fractions can be peptides, in particular, 82 components are in the range of *m*/*z* 500–2000, accounting for 59% of total components, should be small peptides. Accordingly, a majority of the venom components may be small peptides, which are the subject of structural determination as follows.

#### 2.1.2. Identification of Small Molecules (Amino Acids, Biogenic Amines and Nucleic Acids)

As summarized in Table 2, Table 3 and Table 4, 13 amino acids, 5 biogenic amines, and 7 nucleic acids were identified. The identification was made by the previously reported method based on elemental composition analysis of molecular ion (M + H)^+^ with an error limit of 0.005 Da [19,28]. Concomitant detection of iminium ion and deamination (-NH_3_) peak in some case of amino acids and biogenic amines, respectively, was supportive and useful. For nucleic acids (AMP, ADP, and NAD), MS/MS spectra were obtained by data-dependent MS/MS measurement, which confirmed the structure of these compounds.

#### 2.1.3. Peptide Sequencing by MS/MS Analysis

Peptide sequences were manually analyzed their MS/MS spectra obtained by data-dependent MS/MS measurement, which revealed the full sequence of 44 small peptides. The analyzed full sequences are shown in Table 5. These small peptides can be classified according to structural similarity as shown in Table 6. They are broadly divided into two classes: bradykinin-related peptides, and linear α-helical peptides. In each class, there are peptides seemed to be truncated from N- and C-terminus of the “parent peptide” (the longest sequence). It is not sure whether they are originally contained in the venom or cleavage products in some way.

The bradykinin-related peptides can be further divided into three subclasses. The first is Thr^6^-BK and its close relatives. As mentioned in the Introduction section, we already reported the identification of Thr^6^-BK in this venom, but it was only detection of (M + H)^+^ by MALDI-TOF MS [27]. In this study, it was verified by sequence analysis as RPPGFTPFR (*m*/*z* 1074.574). Two closely related peptides were found: Ca-969 (*m*/*z* 969.590, PATLPAPFR: where L = either L or I) and Ca-1056 (*m*/*z* 1056.622, RLPGLTPFR: where L = either L or I). Both of them have the same amino acid length and 3–4 C-terminal sequence as those of Thr^6^-BK, and the rest of N-terminal sequence is different from Thr^6^-BK. The second subclass consists of two peptides: Ca-1365 (*m*/*z* 1365.787, DALPRLLPAPFR: where L = either L or I), and Ca-1452 (*m*/*z* 1365.787, DALPRLLPGTPFR: where L = either L or I). They are quite similar to each other, having the same N-terminal 8 amino acids (DALPRLL), and the C-terminal 3 amino acids (PFR) are the same as Thr^6^-BK. The third subclass includes the two major peptide components: α-campsomerin (*m*/*z* 1534.865, PRLRRLTGLSPLR: where L = either L or I), and β-campsomerin (*m*/*z* 1702.955, PRLRRLTGLSPLRAP: where L = either L or I), which correspond to the two most intense peaks at RT 6.45 min, Fr. 9 and 7.02 min, Fr.10, respectively. They are different each other only at the C-terminal: β-campsomerin has the dipeptide AP at the C-terminal of α-campsomerin. Determination of L (leucine) or I (isoleucine) at the positions 3, 6, 9, and 12 of both peptides was performed by MALDI TOF/TOF MS analysis. The MALDI TOF/TOF spectrum of α-campsomerin afforded d-ion peaks at *m*/*z* 297.3 (d_3_), 722.6 (d_6_), and 993.7(d_9_), and w-ion peaks at *m*/*z* 229.3 (w_2_) and 526.4 (w_5_), which clearly showed that all these residues are L (leucine), not I (isoleucine). Similarly, all the L/I residues were determined as L by MALDI TOF/TOF spectral analysis of β-campsomerin, observing d-ion peaks at *m*/*z* 297.1 (d_3_), 722.3 (d_6_), 993.4 (d_9_), and 1290.8 (d_12_), and w-ion peaks at *m*/*z* 398.1 (w_4_) and 694.3 (w_7_). Finally, solid-phase synthesis of these peptides, and the HPLC and MS/MS comparisons of the synthetic specimens with the natural peptides corroborated the sequences. The C-terminal sequence, GLSPL, of these peptides is similar to that, GFSPL, of Cd-146, α-scoliidine, β-scoliidine, bradykinin-related peptides already known in solitary wasp venoms (Table 7).

**Figure 2:**
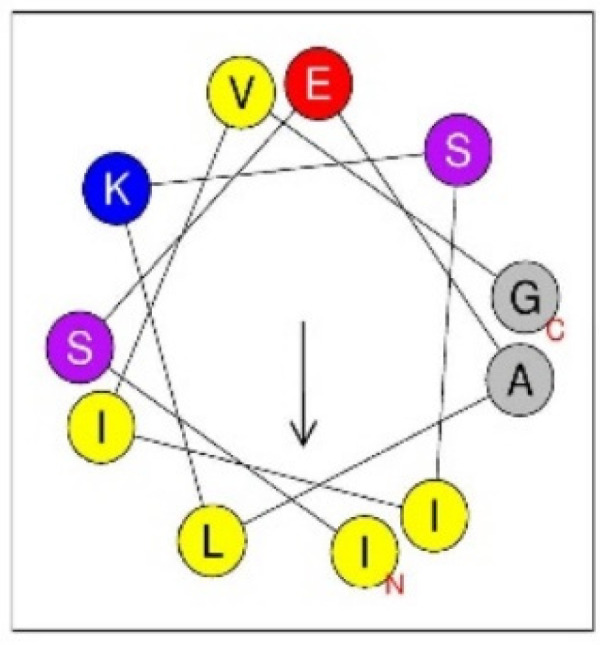
Helical wheel projection of the sequence of annulatin (ISEALKSIIVG-NH_2_). In this view through the helix axis, the hydrophilic Ser (S), Glu (E), and Lys (K) residues are located on one side and the hydrophobic Ala (A), Ile (I), and Leu (L) residues on the other side of the helix.

The third most intense, but rather minor peak at RT 8.57, Fr. 13 contained annulatin (*m*/*z* 1128.687, LSEALKSLLVG-NH_2_: where L = either L or I). The MALDI TOF/TOF spectrum of annulatin afforded d-ion peaks at *m*/*z* 786.2 (d_8a_), 800.2 (d_8b_), and 913.3 (d_9b_), and w-ion peaks at *m*/*z* 669.2 (w7) and 1083.6 (w_11a_), which indicated 1I, 7L, 8I, and 9I. Accordingly, the exact structure should be ISEALKSIIVG-NH_2_. It was confirmed by solid-phase synthesis of this peptide, and the HPLC and MS/MS comparisons of the synthetic specimens with the natural peptide. This peptide may belong to linear α-helical peptides since it can adopt amphiphilic α-helical secondary structure as shown in Figure 2. A large number of this type of peptides are found in natural sources, including arthropod venoms. In case of solitary wasp venoms, we already discovered several peptides of such a type (Table 8), but this is the first example found in scoliid wasp venom. Two other peptides, Ca-1281 (*m*/*z* 1281.875, FLLPLLKGLLVG-NH_2_: where L = either L or I) and Ca-1415 (*m*/*z* 1415.896, GLLTDLRKFLLK-NH_2_: where L = either L or I) may also be linear α-helical peptides, as they can be predicted to adopt α-helical secondary structure.

The rest of the peptides included in the Miscellaneous section are not classified into any groups since they have no similarity to bradykinin-related peptides, linear α-helical peptides, nor any known peptides. Accordingly, chemical and biological characteristics of these peptides are not known.

### 2.2. Biological Characterization of α-Campsomerin and β-Campsomerin

#### 2.2.1. Interaction with NEP and ACE

NEP and ACE catalytic activity was not inhibited by either peptide. Furthermore, neither peptide was broken by the metallopeptidases, indicating that they do not interact with the metallopeptidases under investigation (Table 9).

#### 2.2.2. AChE Activity

**Figure 3:**
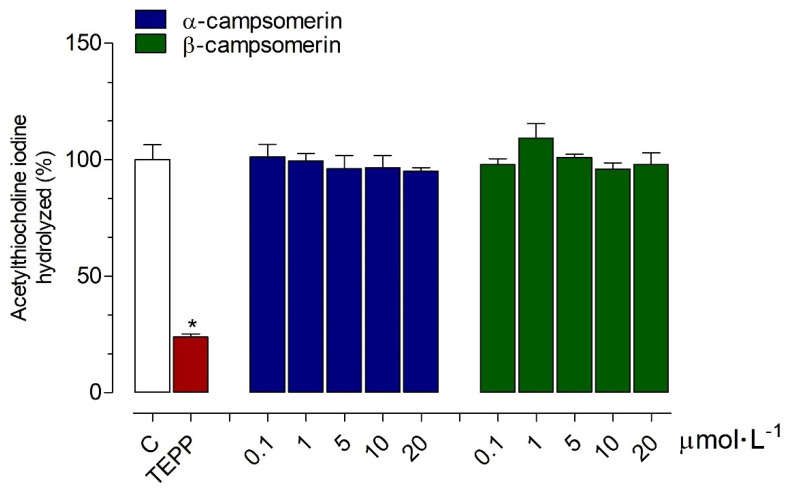
Inhibitory effects of α-campsomerin and β-campsomerin on the activity of acetylcholine esterase (AChE). The extent of AChE activity inhibition was expressed as a percentage of acetylthiocholine iodine substrate hydrolyzed in relation to control (C; blank box). Data from three independent experiments in triplicate are expressed as mean ± standard deviation, and evaluated using one-way ANOVA followed by Tukey’s post-test. * *p* < 0.05 for differences between the control groups; Tetraethyl pyrophosphate (TEPP; red box).

The α-campsomerin and β-campsomerin were tested for AChE inhibitory activities in vitro compared to the tetraethyl pyrophosphate (TEPP; positive control), and untreated AChE reaction (negative control). Both peptides did not exhibit anti-AChE activities compared to the negative control. However, TEPP reduced the AChE activity with an inhibition percentage of 76.20 ± 1.42% (Figure 3).

#### 2.2.3. Cytotoxic Effects

**Figure 4:**
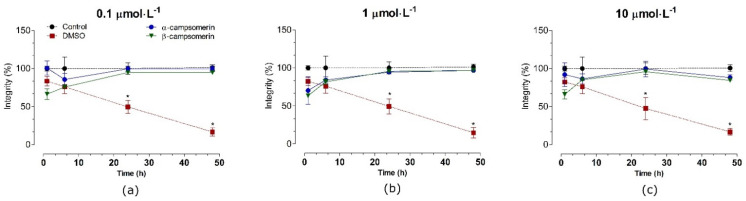
Toxicity of α-campsomerin or β-campsomerin on cell integrity in Vero cells. Cells were treated with peptides 0.1 μmol·L^−1^ (**a**), 1 μmol·L^−1^ (**b**) and 10 μmol·L^−1^ (**c**) for 1, 6, 12, 24, and 48 h. Control and DMSO groups represent cells without treatment and treated with DMSO 5%, respectively. Values are expressed as mean ± SD (*n* = 3 in triplicate) and analyzed by one-way ANOVA followed by Tukey’s post-test. * *p* < 0.05 in relation to the control group.

The cytotoxic effects of α-campsomerin and β-campsomerin were assessed in two different cell types: a typical fibroblastic cell (Vero cells,) and a neuronal cell line (PC12 cells). Both peptides showed no significant cytotoxicity (*p* > 0.05) in Vero cells after 1, 6, 24, and 48 h of treatment when compared to their respective control groups (untreated cells) at all doses tested (Figure 4).

**Figure 5:**
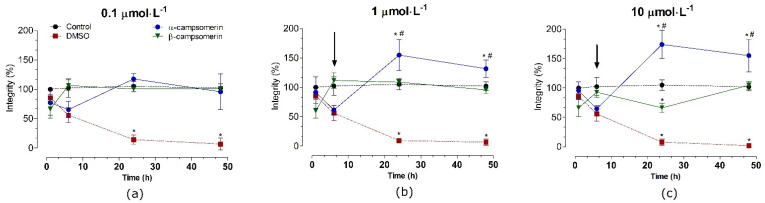
Toxicity of α-campsomerin and β-campsomerin on PC12 cell integrity. Cells were treated with peptides 0.1 μmol·L^−1^ (**a**), 1 μmol·L^−1^ (**b**) and 10 μmol·L^−1^ (**c**) for 1, 6, 12, 24, and 48 h. Control and DMSO groups represent cells without treatment and treated with DMSO 5%, respectively. Data were obtained from three independent experiments in triplicate, expressed as mean ± SD, and analyzed by one-way ANOVA followed by Tukey’s post-test. * *p* < 0.05 compared to the control group; # *p* < 0.05 compared to the β-campsomerin. Arrows indicate the integrity cells reduced by α-campsomerin, but not β-campsomerin, at 6 h of treatment.

In PC12 cells, α–campsomerin reduced cell integrity after 6 h of treatment in all concentrations tested (Figure 5). In spite of that, cells integrity increased significantly (*p* < 0.05) after 24 and 48 h of treatment with this peptide, especially at 1 and 10 µmol·L^−1^, in relation to the control and β–campsomerin groups. When compared to the untreated cell (control) or DMSO, the β-campsomerin showed cytotoxic effects at 10 µmol·L^−1^ after 24 h of treatment (Figure 4). DMSO (positive control) decreased cell integrity in Vero and PC12 cells after 6, 24 and 48 h in comparison to the control group (Figure 4 and Figure 5).

#### 2.2.4. Neuroprotective Effects

**Figure 6:**
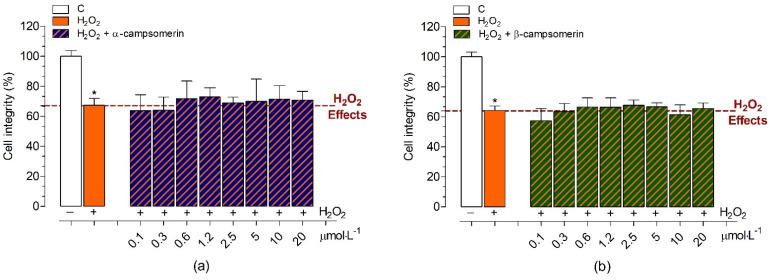
Neuroprotective property of α-campsomerin (**a**) and β-campsomerin (**b**) on cell integrity of the PC12 cell line against H_2_O_2_-induced oxidative stress. PC12 cells were plated at 2 × 10^4^ cells per well in a 96-well plate, and were pre-treated with peptides for 4 h at 37 °C. After that, the medium was replaced containing peptide and H_2_O_2_ 0.5 mmol·L^−1^) and incubated for more 20 h. Data are expressed as the mean ± standard deviation from three independent experiments in triplicate and analyzed using one-way ANOVA followed by Dunnett’s post-test. * *p* < 0.05 for differences between the control and experimental groups.

The neuroprotective effects against H_2_O_2_-induced oxidative stress in the presence of α-campsomerin or β-campsomerin under different concentrations were studied in PC12 cells (Figure 6). Initially, we evaluated the chronic cytotoxic effects promoted by H_2_O_2_ (concentrations varying between 1–0.06 mmol·L^−1^) on PC12 cells after 20 h of treatment. H_2_O_2_ promoted cell death at concentrations greater than 0.3 mmol·L^−1^ in a dose-dependent manner. The 0.5 mmol·L^−1^ concentration of H_2_O_2_ reduced 60.5 ± 4.0% of cell integrity and it was chosen for studies of protective effects of α-campsomerin or β-campsomerin against oxidative stress-induced neurotoxicity. Our results demonstrated that both peptides did not show neuroprotective effects against the H_2_O_2_-induced damage in PC12 cells after chronic treatment (Figure 6).

### 2.3. Antimicrobial, Hemolytic, and Histamine-Releasing Activities of Annulatin and Related Peptides

Annulatin displayed no antimicrobial activities against all the microbes tested in this study, low hemolytic activity, and comparable histamine-releasing activity with mastoparan, famous wasp peptides, in a dose-dependent manner (Table 10). Since many linear α-helical antimicrobial peptides are basic peptides, it is assumed that the lower pI of annulatin displayed no antimicrobial activity. The hydrophobicity of annulatin (0.69) is lower than that of the highest hydrophobic region in melittin (0.88), honeybee hemolytic peptide. Previous studies suggested a correlation between peptide hydrophobicity and hemolytic activity [33].

## 3. Discussion

We have studied venom components of solitary hunting wasps with a special interest in the neuroactive substances due to their functional role, which is to paralyze the prey (spiders and insects). Initially, and until recently, studies were conducted by the conventional way: HPLC purification, followed by chemical characterization of the isolated compounds [15,19,21,22,29,30,31,32]. In this way, however, only a few major components were successfully characterized, despite the large numbers present in the venom. Furthermore, for this, a significant amount of venom extracts is required. This means a collection of a number of individuals at least 20–30 insects, which is usually very difficult because of the lifestyle of solitary wasps. The remarkable progress of mass spectrometry in sensitivity and resolution made it possible to accomplish this type of analysis with a minute amount of venom. Indeed, we reported comprehensive analysis of venom components of solitary scoliid wasp Scolia ventralis using only 10% of the amount of a single venom contents [28].

This highly efficient analytical means was used again for this study. Comprehensive analysis of the *Campsomeriella annulata annulata* venom extract by using LC-ESI-MS attained the component profile, consisting of 138 molecules. Peptide sequences were manually analyzed their MS/MS spectra obtained by data-dependent MS/MS measurement, which revealed the full sequence of 44 small peptides. They are broadly classified into two major structural types: bradykinin-related peptides, and linear α-helical peptides. The bradykinin-related peptides can be further divided into three subclasses. The first one has a high similarity to bradykinin, and the others are only partially similar to the three C-terminal amino acids of bradykinin. Three species of scoliid wasp venoms have so far been studied, and all of these have bradykinin-related peptides [24,25,27,28]. Accordingly, bradykinin-related peptides are common components of scoliid wasp venoms. Another class of peptides, linear α-helical peptides, have mostly been found in eumenine wasp venoms, and this is the first time that such peptides were found in scoliid wasp venoms. With this comprehensive analysis, small molecules (amino acids, biogenic amines, and nucleic acids) could also be identified as previously reported [19,28]. Some of them were reported to be present and functional in solitary wasp venoms. Histamine and tyramine play a role in pain-producing activity [35]. Dopamine in the venom of the emerald jewel wasp *Ampulex compressa* is implicated in a unique behavior of its prey, the American cockroach [11]. In this particular venom, however, their functional role is not yet known. Any other small molecules identified in this venom may give physiological effects when injected into the beetle larvae prey, which remains to be studied.

The two main peptide components identified from the *Campsomeriella annulata annulata* solitary wasp venom, α-campsomerin (PRLRRLTGLSPLR) and β-campsomerin (PRLRRLTGLSPLRAP), were studied in two relevant therapeutic targets related to hypertension, and cardiovascular and renal diseases. ACE (EC 3.4.15.1) and NEP (EC 3.4.24.11) are membrane-anchored vasopeptidases important in blood pressure control. While ACE produces angiotensin II from the hydrolysis of angiotensin I and inactivates bradykinin, NEP degrades natriuretic peptides (ANP, BNP and CNP), in addition to bradykinin and substance P [36]. Interestingly, despite the similarity of both peptides with the C-terminus of bradykinin (RPPGFSPFR), our results demonstrated that they did not behave as substrates or inhibitors of the catalytic activities of NEP and ACE, indicating that they do not interact with the studied metallopeptidases.

Continuing the studies of biological effects of α-campsomerin and β-campsomerin, we investigated the possible interaction of these molecules with AChE (E.C.3.1.1.7). AChE is a well-known serine hydrolase that catalyzes the hydrolysis of the neurotransmitter acetylcholine into choline and acetic acid [37]; inhibiting it would increase acetylcholine levels in the brain, enhancing cholinergic synapses in Alzheimer’s disease patients [38,39]. Natural peptides have garnered a lot of attention as AChE inhibitors [38], but in our study, both peptides did not exhibit anti-AChE activities, as seen by TEPP, an organophosphate pesticide that induces excessive stimulation of the central nervous system leading to respiratory failure and death by irreversibly inhibiting AChE [40].

The venom of solitary wasps is a rich source of neuroactive chemicals [7], including bradykinin-related peptides, which disrupt the synaptic transmission of the nAChR in the central nervous system of prey [24,25,26]. Our group has studied the neuroprotective effects of natural peptides from solitary wasp venoms against the H_2_O_2_-induced oxidative stress in neuronal cell lines after different treatments for acute and chronic conditions. [28]. This cell model, which promotes excessive ROS generation [41,42], with consequent neurotoxic effects [43,44], represents typical characteristics of different neurodegenerative diseases. In a previous study, the neuroprotective effects of the two main peptides α-scoliidine (DYVTVKGFSPLR) and β-scoliidine (DYVTVKGFSPLRKA) identified from the *Scolia decorata ventralis* venom, with significant similarity between the C-terminal of campsomerins, showed that small structural differences of natural peptides can result in significant differences in biological activities [28]. β-scoliidine, but not α-scoliidine, showed neuroprotective effects against H_2_O_2_-induced neurotoxicity in prolonged therapy, preserving neuronal cell integrity and mitochondrial metabolism [28]. It is worth noting that the effects of β-scoliidine were only reliant on the presence of two extra amino acid residues (KA) at the C-terminal on its main molecular sequence [28], and this would explain its protective effects against oxidative stress-induced neurotoxicity in these cells. In the present study, we also investigated the neuroprotective effects against H_2_O_2_-induced stress in the presence of α-campsomerin or β-campsomerin, at different concentrations, in PC12 neuronal cells. Interestingly, despite the relative similarity between the C-terminal of campsomerins and scoliidines, these peptides did not show neuroprotective effects against damage induced by oxidative stress after chronic treatment.

Cytotoxicity assays evaluate the effects of a compound on different mammalian cell lines, providing vital information on the biological characteristics and basic tolerance of a new molecule—an important aspect of the modern pharmaceutical development process [45]. Here, the cytotoxic effects of α-campsomerin and β-campsomerin were studied using two different cell types: a typical fibroblastic cell (Vero cells), and a neuronal PC12 cell line. Both peptides were not cytotoxic in Vero cells in concentrations up to 10 µmol.L^−1^ after 48 h of incubation, in contrast to the DMSO, which reduced cell integrity, in agreement with the literature [46]. In PC12 cells, β-campsomerin was also not toxic under the conditions tested, but α-campsomerin increased the number of cells after 24 to 48 h of treatment. Different biological effects of natural components on cell damage or toxicity in tumor (PC12, HepG2, Caco-2 and 4T1) and non-tumor (Vero, CHO, 3T3, MCDK and BHK2) cell lines have been reported in the literature [47,48]. *Pseudocerastes* venoms—desert snakes of the Viperidae family—reduced the viability of the tumor cells, while having a limited effect on healthy cells, showing the specificity of their effects on tumor cells [47]. Interestingly, we also found that the α-campsomerin increased the viability of neuronal PC12 cells by more than 50% when compared to the β-campsomerin, while it had essentially no effect on the typical fibroblasts (Vero cell line). These results suggest that α-campsomerin appears to stimulate cell viability up-regulation (mitogenic agent) in tumoral cells. It is important to note that the only difference between the primary structures from the studied peptides is the presence of the AP extension at the C-terminus of β-campsomerin compared to α-campsomerin, indicating, again, that small structural differences of natural peptides lead to different biological activities. Hence, for these reasons, more research is needed to understand the potentiating effects of cell viability in the presence of α-campsomerin.

Annulatin is unique as a linear α-helical peptide. Structurally, it has a short length (11 amino acids length), and only one cationic residue (6K) with one anionic residue (3E). Usually, this type of peptide has 2–4 cationic residues (K and R) without any anionic residues, and a length of 10–30 amino acids. In biological activities, linear α-helical peptides commonly show antimicrobial, hemolytic, and histamine-releasing activities [20,29,30,31,32]. Annulatin also showed histamine-releasing activity and weak hemolytic activity, but no antimicrobial activity. Linear α-helical peptides are present in many arthropod venoms, such as scorpion and spider venoms [49,50]. They may be involved in functional roles in preventing the prey from microbial infection during long-time storage, and potentiating venom toxicity by disturbing excitable membranes [49,50]. It is the case for those in solitary wasp venoms, as we have reported previously [20,29,30,31,32] (Table 8). However, in this annulatin case, there is no antimicrobial activity, and accordingly, it may have some other role in venom function, which is a subject of further study. In this sense, future in silico analysis using platforms to predict the possible antihypertensive and antimicrobial activities of all peptides present in *C. annulata annulata* venom are being carried out to define the next molecules to be synthesized.

## 4. Conclusions

Component profile of the venom from the solitary scoliid wasp *Campsomeriella annulata annulata* was revealed by comprehensive LC-MS and MS/MS analyses. The two major peptide components, α-campsomerin (PRLRRLTGLSPLR) and β-campsomerin (PRLRRLTGLSPLRAP), are bradykinin-related peptides. α-Campsomerin increased the viability of neuronal PC12 cells, whereas β-campsomerin had no effect. A minor peptide component annulatin (ISEALKSIIVG-NH_2_) is a linear α-helical peptide with a unique biological activity profile, showing histamine-releasing activity from mast cells. This is the first case in which linear α-helical peptides were found in scoliid wasp venoms.

## 5. Materials and Methods

### 5.1. Materials

All chemicals used in the present study were of analytical reagent grade (purity higher than 95%) and purchased from Calbiochem-Novabiochem Corporation (San Diego, CA, USA), Gibco BRL (New York, NY, USA), Fluka Chemical Corp. (Buchs, Switzerland) or Sigma-Aldrich Corporation (St. Louis, MO, USA). The ACE I from rabbit lung and AChE from Electrophorus electricus (electric eel) Type VI-S were purchased from Sigma-Aldrich. Neprilysin and the Fluorescence Resonance Energy Transfer (FRET) substrates, Abz-FRK (Dnp) P-OH (for ACE I assays) and Abz-RGFK (Dnp)-OH (for NEP assays) were provided by Prof. Adriana Carmona, from the Department of Biophysics of UNIFESP-EPM, São Paulo, SP, Brazil. For the reverse phase chromatography, acetonitrile and TFA were acquired from J.T. Baker.

### 5.2. Wasp Collection

For this study, five female wasp individuals of *Campsomeriella annulata annulata* were collected manually by an insect-catching net in Kyoto, Japan, in August 2010. The venom sacs were dissected under a low temperature anesthetization and extracted with 50% MeCN (acetonitrile)/water. The extracts were lyophilized and stored at −35 °C until use.

### 5.3. Cell Lines

Two types of cell line were used in the present study: PC12 cells (ATCC^®^ CRL-1721™ from the American Type Culture Collection—ATCC, Manassas, VA, USA) was obtained from a transplantable rat pheochromocytoma, and Vero cells (*Cercopithecus aethiops*—CCIAL 057, provided by the Adolfo Lutz Institute, São Paulo, Brazil) from kidney cells of the African green monkey. PC12 cells were cultured in DMEM medium (Sigma-Aldrich), while the Vero cells were maintained in Ham-F-10 medium (Sigma-Aldrich, St. Louis, MO, USA). Both cell cultures were supplemented with 10% fetal bovine serum (FBS; Gibco, Waltham, MA, USA), 1% (*v*/*v*) of penicillin (10,000 U·mL^−1^), streptomycin (10 mg·mL^−1^), and amphotericin B solution (25 μg·mL^−1^) (Sigma-Aldrich, St. Louis, MO, USA). The cultures were kept at 37 °C in a humidified atmosphere containing 5% CO_2_ and 95% air (Water Jacketed CO_2_ Incubator, Thermo Scientific). Cells were passaged using trypsin-EDTA solution [0.05% (*m*/*v*) trypsin and 0.02% (*m*/*v*) EDTA] at 80 percent confluence after the culture medium was replenished every 2–3 days.

### 5.4. LC-ESI-MS

The crude venom was analyzed with a LC (Accela 600 Pump, Thermo Scientific) connected with ESI-FTMS (LTQ Orbitrap XL, Thermo Scientific). The lyophilized venom sac extracts were dissolved into 500 μL of water, and, from this solution, 10 μL (corresponding to 10% amount of crude venom sac extracts from a single specimen) was subjected to reversed-phase HPLC using CAPCELL PAK C_18_ UG 120, 1.5 × 150 mm (SHISEIDO Co., Ltd., Tokyo, Japan) with linear gradient from 5% to 65% CH_3_CN/H_2_O/0.1% (*v*/*v*) formic acid at a flow rate of 200 µL·min^−1^ over 20 min at 25 °C. ESI-FTMS was operated by Xcalibur^TM^ software (Thermo Scientific) as: capillary voltage, +4.6 kV; capillary temp., 350 °C; sheath and aux gas flow, 50 and 30, respectively (arbitrary units); resolution, 5 ppm. MS/MS spectra were obtained by data-dependent MS/MS mode (the two most intense peaks by HCD), and the obtained spectra were manually analyzed to give peptide sequences, which were confirmed by MS-Product in ProteinProspector program (http://prospector.ucsf.edu/prospector/cgi-bin/msform.cgi?form=msproduct, accessed on 8 November 2021).

### 5.5. MALDI-TOF MS

MALDI-TOF MS spectra were acquired on an Autoflex TOF/TOF mass spectrometer (Bruker Daltonics, Yokohama, Japan) equipped with 337 nm pulsed nitrogen laser under reflector mode. The resolution and accuracy of MS were 18,000 full width at half maximum (*m*/*z* 3000) and 10 ppm, respectively. The accelerating voltage was 20 kV. Matrix, α-cyano-4-hydroxycinnamic acid (Sigma-Aldrich, St. Louis, MO, USA), was prepared at a concentration of 10 mg·mL^−1^ in 1:1 CH_3_CN/ 0.1% (*v*/*v*) TFA. External calibration was performed with [Ile^7^]-angiotensin III (*m*/*z* 897.51, monoisotopic; Sigma, St. Louis, MO, USA ) and human ACTH fragment 18–39 (*m*/*z* 2465.19, monoisotopic; Sigma, St. Louis, MO, USA). The sample solution (0.5 µL) dropped onto the MALDI sample plate was added to the matrix solution (0.5 µL) and allowed to dry at room temperature. For TOF/TOF measurement, argon was used as a collision gas and ions were accelerated at 19 kV. The series of *b* and *y* ions were afforded, which enabled identification of whole amino acid sequence by manual analysis.

### 5.6. Peptide Synthesis

The peptides were synthesized using Fmoc chemistry by GenScript (Nanjing, China). The crude products were purified by RP-HPLC with a preparative C18 column, and the purity and molecular weight of the final peptides were verified by HPLC and MS.

### 5.7. ACE and NEP Activities

Experiments were performed using different concentrations of α-campsomerin and β-campsomerin with ACE and NEP, and their FRET substrates, FRK(Dnp)P-OH and Abz-RGFK (Dnp)-OH, respectively. The assays used 7.5 ng of both peptidases and the substrates were added in a 100 mmol·L^−1^ Tris-HCl buffer containing 50 mmol·L^−1^ NaCl, 10 μmol·L^−1^ ZnCl_2_, pH 7.0 (for ACE assays) and Tris HCl 50 mmol·L^−1^, pH 7.5 buffer (for NEP assays). All experiments were carried out at 37 °C and at a final volume of 100 µL Three FRETs substrate concentrations were used (2 μmol·L^−1^, 4 μmol·L^−1^ and 8 μmol·L^−1^), and were incubated with three concentrations of both peptides (20 μmol·L^−1^, 30 μmol·L^−1^ and 50 μmol·L^−1^). Controls without the peptides were also performed in all assays. The reactions were monitored for 15 min on fluorimeter (Victor 3—Perkin–Elmer) and the results were analyzed on GraFit 3.0 from Erithacus Software. All assays were performed in triplicate.

### 5.8. Stability Tests of Peptides

Both peptides (30 µmol·L^−1^) were incubated for four hours at 37 °C with ACE and NEP (7.5 *n*). As a negative control, samples containing only synthetic peptides were employed. After incubation, samples were analyzed on a Shim-pack VP-ODS C-18 column (4.6 150 mm) utilizing reverse-phase chromatography on HPLC (Prominence, Shimadzu). Solvent A was 0.1% TFA in water (solvent A), and solvent B was acetonitrile plus solvent A (9:1). Over a 20-min period, separations were carried out at a flow rate of 1 mL/min with a 10–60 percent gradient of solvent B. Elution was always followed by an assessment of UV absorption (214 nm).

### 5.9. Measurement of AChE Activity

The AChE activity was evaluated spectrophotometrically using Ellman’s technique, as previously reported by Harry et al. [51], with slight modifications. Both peptides (20 μL) at varied concentrations (20, 10, 5, 1, and 0.1 µmol·L^−1^), 10 μL of AChE (0.5 U·mL^−1^) (Sigma, St. Louis, MO, USA) and 160 μL of 5,5-dithiobis-2-nitrobenzoic acid (DTNB, Ellman’s reagent) 0.33 mM (Sigma, St. Louis, MO, USA) in phosphate buffer (0.1 mo·L^−1^, pH 8.0) were placed in triplicate in a microplate, and incubated at room temperature for 10 min after adding 10 μL of acetylthiocholine iodide (20 mmol·L^−1^) (Sigma, St. Louis, MO, USA). The hydrolysis of acetylthiocholine iodide leads to production of acetic acid and thiocholine, which reacts with DTNB producing the anion 5-thio-2-nitrobenzoic acid, which was monitored at 412 nm during 20 min in microplate reader (BioTek Instruments, Inc., Winooski, VT, USA. Tetraethyl pyrophosphate (TEPP; Sigma, St. Louis, MO, USA) was used as a positive control. IAChE = [(Ac − As)/Ac] × 100, where Ac = absorbance for the control and As = absorbance for the sample was used to compute the percentage of inhibition (I) of AChE.

### 5.10. Toxicity Studies on the Integrity Cell

The cytotoxic effects of α-campsomerin and β-campsomerin were determined by the staining of attached cells with crystal violet dye, according to the literature [52]. In a 96-well plate (Nest Biotechnology, Rahway, USA), PC12 and Vero cells were put at 2.0 × 10^4^ and 1.0 × 10^4^ cells/well, respectively. Cells were treated with different concentrations (0.1 to 10 µmol·L^−1^) of peptides in 0.10 mL. The plate was incubated for 1, 6, 24, and 48 h at 37 °C. There were control and DMSO groups for each concentration and time course tested, representing untreated cells (just one equal volume of culture medium) and cells treated with DMSO (5%; *v*/*v*) diluted in the medium culture, respectively. The media was then aspirated, and the cells were stained with a 0.5 percent crystal violet staining solution (0.5%; *m*/*v*), washed, and air-dried. Afterwards, methanol (200 μL) was added, and the absorbance measured at 570 nm using a SpectraMax reader (Molecular Devices, CA, USA). Data were obtained from three independent experiments in triplicate, and were expressed by percentage of cell viability, calculated using the control group as 100% of integrity.

### 5.11. Neuroprotective Assay

Initially, PC12 cells were seeded at 2 × 10^4^ cells/well in a 96-well plate (Nest Biotechnology, Rahway, NJ, USA) for 24 h. To evaluate the neuroprotective effects of peptides, cells were pre-treated at 37 °C with α-campsomerin and β-campsomerin (0.1 to 20 µmol·L^−1^), diluted in DMEM medium supplemented with antibiotics without fetal bovine serum for 4 h. Afterwards, the solutions were replaced by medium containing the peptides and H_2_O_2_ (0.5 mmol·L^−1^) and incubated for 20 h more. Cells untreated with H_2_O_2_ represent control group in all experiments. Next, all groups were analyzed by the cell integrity.

### 5.12. Antimicrobial, Hemolytic, and Histamine Releasing Activities

Antimicrobial, hemolytic, and histamine-releasing activities were measured as described previously [53]. The microbial species used in this study were: Escherichia coli (NBRC 14237), Staphylococcus aureus (NBRC 12732), and Saccharomyces cerevisiae (NBRC 10217), which were purchased from the Biological Resource Center (BRC, Kisarazu, Japan), National Institute of Technology and Evaluation. The pI of peptides was calculated with IPC (http://isoelectric.org/index.html, accessed on 8 November 2021). The physicochemical properties were examined using HeliQuest (http://heliquest.ipmc.cnrs.fr/, accessed on 8 November 2021).

### 5.13. Statistical Analyses

All data are shown in triplicate as the mean ± SD of three different experiments (*n* = 3). For between-groups comparisons, one-way analysis of variance (ANOVA) was used, followed by a Tukey’s post-hoc test for multiple comparisons. Statistical significance was defined as a value of *p* < 0.05. GraphPad Prism 6.0 (GraphPad Software, Inc., La Jolla, CA, USA) was used to conduct the analysis.

## Figures and Tables

**Table 1:** On-line mass fingerprinting of crude venom extract from *Campsomeriella annulata annulata* by LC-ESI-MS.

Fr. No.	Retention Time (min)	[M + H]^+^ *m*/*z*
1	0.8–1.5	90.054, 112.086, 116.069, 118.085, 120.064, 146.164, 147.075, 148.059, 154.085, 156.076, 175.118, 203.222, 258.108, 752.482
2	1.5–2.0	132.101, 138.090, 150.057, 182.080, 245.075, 253.116, 268.102, 284.097, 324.126, 348.067, 664.111
3	2.0–3.0	166.085, 269.086, 732.288, 873.461, 911.428, 916.637, 952.596, 1006.514, 1050.563, 1377.700, 1629.831
4	3.0–4.0	272.133, 660.387, 911.497, 953.580, 1164.582, 1435.706, 1487.628
5	4.0–5.0	205.095, 573.309, 585.367, 642.388, 1085.694, 1227.626, 2741.410, 2913.678, 2927.693, 2941.668, 2981.607, 3025.066
6	5.0–5.6	497.400, 771.408, 939.662, 1148.716, 1423.836, 2218.047
7	5.6–6.1	378.163, 487.319, 728.497, 1418.844, 1439.702, 1548.844, 5297.517, 6211.754
8	6.1–6.3	403.251, 555.356, 656.346, 1074.574, 1127.483, 1265.684, 1447.679, 1719.983
9	6.3–6.8	753.455, 969.590, 1105.569, 1534.865, 1587.776, 2068.808, 2229.234, 3251.858
10	6.8–7.1	419.237, 638.392, 1056.622, 1247.703, 1702.955
11	7.1–7.7	1060.438, 1378.768, 2208.189, 3015.057, 3251.857
12	7.7–8.3	918.474, 1499.748, 3152.777, 4089.223
13	8.3–8.6	900.542, 1128.687, 1182.607, 1250.760, 1266.756, 1500.731, 2244.169, 4716.118
14	8.6–9.0	813.492, 1072.654, 1258.713, 2913.497
15	9.0–10.0	1365.787, 1452.818, 1902.932, 2765.478
16	10.0–11.0	918.557, 995.474, 1281.875, 1415.896, 1840.016, 1893.933
17	11.0–11.8	1274.625, 1593.932, 1645.945, 1689.987, 3640.506
18	11.8–12.3	1761.009, 1991.029, 2043.940, 2091.104, 2291.182
19	12.3–12.7	1324.985, 1689.951
20	12.7–13.8	1317.740, 1518.891, 1856.998, 1909.904

**Table 2:** Amino acids in the crude venom extract from *Campsomeriella annulata annulata* by LC-ESI-MS.

RT (min)	Intensity × 10^4^	[M + H]^+^ *m*/*z*	Elemental Composition	Iminium Ion *m*/*z*	Elemental Composition	Compound
1.07	50	156.076	C_6_H_10_N_3_O_2_	—		Histidine
	140	175.118	C_6_H_15_N_4_O_2_	—		Arginine
1.28	24	90.054	C_3_H_7_NO_2_	—		Alanine
	18	120.064	C_4_H_10_NO_3_	—		Threonine
	180	147.075	C_5_H_11_N_2_O_3_	—		Glutamine
	200	148.059	C_5_H_10_NO_4_	102.054	C_4_H_8_NO_2_	Glutamic acid
1.34	700	116.069	C_5_H_10_NO_2_	70.064	C_4_H_8_N	Proline
	70	118.085	C_5_H_12_NO_2_	72.080	C_4_H_10_N	Valine
1.55	13	150.057	C_5_H_12_NO_2_S	104.052	C_4_H_10_NS	Methionine
1.68	180	132.101	C_6_H_14_NO_2_	86.096	C_5_H_12_N	L/I *
1.72	80	182.080	C_9_H_12_NO_3_	—		Tyrosine
2.37	180	166.085	C_9_H_12_NO_2_	120.080	C_8_H_10_N	Phenylalanine
4.17	13	205.096	C_11_H_13_N_2_O_2_	—		Tryptophan

* Either leucine (L) and/or isoleucine (I).

**Table 3:** Biogenic amines in the crude venom extract from *Campsomeriella annulata annulata* by LC-ESI-MS.

RT (min)	Intensity × 10^4^	[M + H]^+^ *m*/*z*	Elemental Composition	Deamination *m*/*z*	Elemental Composition	Compound
1.01	7	146.164	C_7_H_20_N_3_	—		Spermidine
1.07	12	112.086	C_5_H_10_N_3_	95.060	C_5_H_7_N_2_	Histamine
	6	203.222	C_10_H_27_N_4_	—		Spermine
1.34	12	154.086	C_8_H_12_NO_2_	137.059	C_8_H_9_O_2_	Dopamine
1.61	700	138.090	C_8_H_12_NO	121.064	C_8_H_9_O	Tyramine

**Table 4:** Nucleic acids in the crude venom extract from *Campsomeriella annulata annulata* by LC-ESI-MS.

RT (min)	Intensity × 10^4^	[M + H]^+^ *m*/*z*	Elemental Composition	Compound
1.21	10	244.091	C_9_H_14_N_3_O_5_	Cytidine
1.28	180	258.108	C_10_H_16_N_3_O_5_	Thymidine
1.61	100	348.068	C_10_H_15_N_5_O_7_P	AMP
	18	664.111	C_21_H_28_N_7_O_14_P_2_	NAD
1.68	1800	268.102	C_10_H_14_N_5_O_4_	Adenosine
1.72	8	245.075	C_9_H_13_N_2_O_6_	Uridine
1.97	90	284.097	C_10_H_14_N_5_O_5_	Guanosine

**Table 5:** Peptide sequences analyzed from MS/MS spectra.

Fr	RT	Intens. × 10^3^	MSMS *m*/*z*	Charge	(M + H)^+^	Sequence
1	1.23	70	251.499	3+	752.482	SKLHRL-NH_2_
2	1.95	85	253.116	+	253.116	SF
3	2.09	91	459.905	3+	1377.700	RGPRTYSHGHPL
	2.27	1500	318.204	3+	952.596	SLSKLHRL-NH_2_
	2.53	8	304.481	3+	911.428	TYSHGHPL
	2.78	35	366.648	2+	732.288	HNAEFD
4	3.06	8	318.532	3+	953.580	SLSKLHRL
	3.34	72	330.697	2+	660.387	LSEALK
	3.55	330	456.253	2+	911.497	PRLPRLT
	3.66	750	479.240	3+	1435.706	RDPRTYSHGHPL
	3.80	1500	388.865	3+	1164.582	PRTYSHGHPL
5	4.06	42	293.187	2+	585.367	LSPLR
	4.56	350	287.159	2+	573.309	RPPGF
	4.99	60	321.698	2+	642.388	GLSPLR
6	5.18	85	383.577	3+	1148.716	VPSLKSLHRL-NH_2_
	5.37	90	386.208	2+	771.408	RPPGFTP
	5.60	29	313.893	3+	939.662	LVKQKVLL-NH_2_
7	5.79	80	378.163	+	378.163	DFP
	6.00	90	364.751	2+	728.495	LKSLLVG-NH_2_
8	6.07	8	403.251	+	403.251	LTGL
	6.26	600	358.863	3+	1074.574	RPPGFTPFR (Thr^6^-BK)
9	6.41	11	377.231	2+	753.455	RLPGLTP
	6.45	62,000	512.294	3+	1534.865	PRLRRLTGLSPLR
	6.74	860	323.869	3+	969.590	PATLPAPFR
10	6.84	210	416.572	3+	1247.703	RLVKPVPFYE
	6.94	1200	352.879	3+	1056.622	RLPGLTPFR
	7.02	12,000	568.324	3+	1702.955	PRLRRLTGLSPLRAP
11	7.63	85	530.723	2+	1060.438	HNAEFDAAW
	7.67	390	689.887	2+	1378.768	PRLRRLTGLSPL
12	7.80	52	459.741	2+	918.474	RPPGFTPF
13	8.57	2200	564.848	2+	1128.687	LSEALKSLLVG-NH_2_
14	8.72	470	536.829	2+	1072.650	LSEALKSLLV
	8.73	160	407.249	2+	813.490	PATLPAPF
	8.84	460	629.861	2+	1258.713	LSEALKSLLVGE
15	9.07	45	455.934	3+	1365.787	DALPRLLPAPFR
	9.17	31	484.945	3+	1452.818	DALPRLLPGTPFR
	9.70	135	634.982	3+	1902.932	DTFGPLYDKLHQYLGH-NH_2_
16	10.69	16	427.964	3+	1281.875	FLLPLLKGLLVG-NH_2_
	10.81	28	472.637	3+	1415.896	GLLTDLRKFLLK-NH_2_
	10.95	16	459.783	2+	918.557	GLVYLLQL
17	11.72	9	549.320	3+	1645.945	DDGLLTDLRKFLLK-NH_2_
18	11.92	30	697.706	3+	2091.104	HPDDDDDFLLPLLKGLLVG-NH_2_
	12.01	1600	664.348	3+	1991.029	DDDDDGLLTDLRKFLLK-NH_2_
20	13.11	1050	619.669	3+	1856.992	DDDDDFLLPLLKGLLVG-NH_2_

**Table 6:** Classification of the peptide sequences.

RT	Intensity × 10^3^	(M + H)^+^	Sequence
**Bradykinin-related peptides**			
4.56	350	573.309	RPPGF
5.37	90	771.408	RPPGFTP
7.80	52	918.474	RPPGFTPF
6.26	600	1074.574	RPPGFTPFR (Thr^6^-BK)
			
6.41	11	753.455	RLPGLTP
6.94	1200	1056.622	RLPGLTPFR (Ca-1056)
			
8.73	160	813.490	PATLPAPF
6.74	860	969.590	PATLPAPFR (Ca-969)
			
9.07	45	1365.787	DALPRLLPAPFR (Ca-1365)
9.17	31	1452.818	DALPRLLPGTPFR (Ca-1452)
			
3.55	330	911.497	PRLRRLT
7.67	390	1378.768	PRLRRLTGLSPL
6.07	8	403.251	LTGL
4.06	42	585.367	LSPLR
4.99	60	642.388	GLSPLR
6.45	62,000	1534.865	PRLRRLTGLSPLR (α-campsomerin)
7.02	12,000	1702.955	PRLRRLTGLSPLRAP (β-campsomerin)
**Linear α-helical peptides**			
3.34	72	660.387	LSEALK
8.72	470	1072.650	LSEALKSLLV
6.00	90	728.495	LKSLLVG-NH_2_
8.57	2200	1128.687	LSEALKSLLVG-NH_2_ (annulatin)
8.84	460	1258.713	LSEALKSLLVGE
			
10.69	16	1281.875	FLLPLLKGLLVG-NH_2_ (Ca-1281)
13.11	1050	1856.992	DDDDDFLLPLLKGLLVG-NH_2_
11.92	30	2091.104	HPDDDDDFLLPLLKGLLVG-NH_2_
			
10.81	28	1415.896	GLLTDLRKFLLK-NH_2_ (Ca-1415)
11.72	9	1645.945	DDGLLTDLRKFLLK-NH_2_
12.01	1600	1991.029	DDDDDGLLTDLRKFLLK-NH_2_
**Miscellaneous**			
1.23	70	752.482	SKLHRL-NH_2_
2.27	1500	952.596	SLSKLHRL-NH_2_
3.06	8	953.580	SLSKLHRL
5.18	85	1148.716	VPSLKSLHRL-NH_2_
			
2.78	35	732.288	HNAEFD
7.63	85	1060.438	HNAEFDAAW
			
2.53	8	911.428	TYSHGHPL
3.80	1500	1164.582	PRTYSHGHPL
2.09	91	1377.700	RGPRTYSHGHPL
3.66	750	1435.706	RDPRTYSHGHPL
			
1.95	85	253.116	SF
5.79	80	378.163	DFP
10.95	16	918.557	GLVYLLQL
5.60	29	939.662	LVKQKVLL-NH_2_
6.84	210	1247.703	RLVKPVPFYE
9.70	135	1902.931	DTFGPLYDKLHQYLGH-NH_2_

**Table 7:** Bradykinin-related peptides in solitary wasp venoms.

Peptide	Sequence	References
Bradykinin (BK)	RPPGFSPFR	[21,27]
Thr^6^-Bradykinin (Thr^6^-BK)	RPPGFTPFR	[21,24,25,27]
Megascoliakinin	RPPGFTPFRKA	[24]
Cyphokinin	DTRPPGFTPFR	[21]
Fulvonin	SIVLRGKAPFR	[21]
Cd-146	SETGNTVTVKGFSPLR	[21]
α-Scoliidine	DYVTVKGFSPLR	[28]
β-Scoliidine	DYVTVKGFSPLRKA	[28]
α-Campsomerin	PRLRRLTGLSPLR	This work
β-Campsomerin	PRLRRLTGLSPLRAP	This work

**Table 8:** Linear α-helical peptides in solitary wasp venoms.

Peptide	Sequence	References
Anoplin	GLLKRIKTLL-NH_2_	[29]
Decoralin	SLLSLIRKLIT	[30]
Eumenitin	LNLKGLFKKVKSLLT	[31]
Eumenitin-F	LNLKGLFKKVASLLT	[32]
Eumenitin-R	LNLKGLIKKVASLLN	[32]
Annulatin	ISEALKSIIVG-NH_2_	This work

**Table 9:** Hydrolyses of α-campsomerin and β-campsomerin by NEP and ACE.

	Inhibition (%)		Cleavage (%)	
Peptide	NEP	ACE	NEP	ACE
α-campsomerin	<0.01	<0.01	<0.01	<0.01
β-campsomerin	<0.01	<0.01	<0.01	<0.01

**Table 10:** Antimicrobial, hemolytic, and histamine-releasing activities of annulatin and related peptides.

	MIC ^a^ (μmol·L^−1^)			Hemolytic Activity (%)		Histamine Releasing Activity at 10 μmol·L^−1^ (%)
	*E. coli* (NBRC 14237)	*S. aureus* (NBRC 12732)	*S. cerevisiae* (NBRC 10217)			
				at 10 μmol·L^−1^	at 12.5 μmol·L^−1^	
Annulatin	Negative	Negative	Negative	-	15.4	33.0
Mastoparan	-	-	-	13.5 ^b^	-	31.1 ^b^
Melittin	-	-	-	100.0 ^b^	-	64.3 ^b^

^a^ MIC: Minimum Inhibitory Concentration. ^b^ The activities from Shigeri et al. [34] were indicated. -: Not determined.

## Data Availability

All data generated or analyzed during this study are included in this published article.
